# Associations between glutamate and cerebral blood flow in treatment-resistant schizophrenia during clozapine treatment

**DOI:** 10.1177/02698811251409141

**Published:** 2026-01-08

**Authors:** Junyu Sun, Fernando Zelaya, Kyra-Verena Sendt, Grant McQueen, Amy L. Gillespie, John Lally, Owen O’Daly, David J. Lythgoe, Oliver D. Howes, Gareth J. Barker, Philip McGuire, James H. MacCabe, Alice Egerton

**Affiliations:** 1 Department of Psychosis Studies, Institute of Psychiatry, Psychology & Neuroscience, King’s College London, UK; 2 Department of Neuroimaging, Institute of Psychiatry, Psychology & Neuroscience, King’s College London, UK; 3 Department of Psychiatry, Warneford Hospital, University of Oxford, UK; 4 National Psychosis Unit, South London and Maudsley NHS Foundation Trust, UK; 5 Department of Psychiatry, University College Dublin, Ireland; 6 Department of Psychiatry, St Vincent’s Hospital Fairview, Ireland

**Keywords:** Treatment-resistant schizophrenia, clozapine, glutamate, cerebral blood flow

## Abstract

**Background:**

Treatment-resistant schizophrenia (TRS) is associated with alterations in glutamate levels and cerebral blood flow (CBF), and treatment with clozapine appears to have an impact on each of these measures. Here, we examined whether changes in CBF and glutamate levels following clozapine treatment are related.

**Methods:**

Glutamate concentrations in the anterior cingulate cortex (ACC), and striatum (measured using proton magnetic resonance spectroscopy, ^1^H-MRS); and whole brain regional CBF maps (measured using pseudo-continuous arterial spin labelling (pCASL)), were examined in a cohort of TRS subjects before and after 12 weeks of treatment with clozapine (baseline: *N* = 30; week 12: *N* = 20).

**Results:**

The longitudinal change in striatal glutamate during treatment was positively associated with the longitudinal change in striatal CBF (*p* < 0.05). In the ACC, higher Glx (glutamate + glutamine) and CBF prior to treatment were associated with greater subsequent improvement in total symptom severity.

**Conclusions:**

These results indicate that there is a direct relationship between changes in glutamate activity and CBF in the striatum following clozapine treatment. Future research could investigate whether glutamate-CBF relationships exist in other stages of psychosis or schizophrenia and during treatment with other antipsychotic medications. Associations with clinical outcomes could be explored in larger samples.

## Introduction

Approximately a third of patients with schizophrenia do not respond to standard antipsychotic medications and are hence categorised as having treatment-resistant schizophrenia (TRS) (Howes et al., 2017). Clozapine is considered the gold standard treatment for TRS due to its superior efficacy in this patient population (The Lancet Psychiatry, 2024). However, the neurobiological basis of TRS and the mechanism of action of clozapine remain unclear.

Schizophrenia is associated with alterations in both brain glutamate concentrations (Egerton et al., 2020) and cerebral blood flow (CBF; Goozée et al., 2014). Recent systematic reviews and meta-analyses have indicated higher Glx (glutamate plus glutamine) in the basal ganglia across the schizophrenia-spectrum disorders (Merritt et al., 2016, 2023; Nakahara et al., 2022), lower medial frontal cortex glutamatergic levels with individual differences in the degree of glutamate dysfunction (Merritt et al., 2023), and cortical hypoperfusion and subcortical hyperperfusion (Percie du Sert et al., 2023). Neurobiological alterations in brain glutamate and blood flow might be more prominent in TRS. For example, in the meta-analysis by Nakahara et al. (2022), while there were no significant alterations in frontal cortical glutamate metabolites overall in patients compared to controls, the TRS group exhibited higher glutamate and Glx in the mid-cingulate cortex, though this effect was not seen in the basal ganglia. We have also observed widespread reductions in CBF in TRS compared to control, across cortical (anterior cingulate cortex (ACC)) and subcortical (hippocampus, thalamus, & striatum) regions (Sun et al., 2024).

Alterations in glutamate concentration and local CBF are thought to be functionally related via neurovascular coupling. As the primary excitatory neurotransmitter, glutamate plays a crucial role in regulating local CBF to meet metabolic demands (Attwell et al., 2010). Glutamate release and activation of N-methyl-D-aspartate (NMDA) receptors on astrocytes evoke increases in intracellular Ca^2+^ and release of vasoactive substances to cause local vasodilation (Attwell et al., 2010; Fergus and Lee, 1997; Zhang et al., 2024). While there are few human brain *in vivo* studies examining local associations between glutamate and CBF, findings in healthy volunteers include a positive correlation between pre-supplementary motor area Glx and local grey matter CBF (Krishnamurthy et al., 2022), and between ACC glutamate concentration and ACC grey matter CBF, which was disrupted on administration of the NMDA antagonist ketamine (Bojesen et al., 2018). In individuals with a diagnosis of schizophrenia, a positive correlation between ACC glutamate concentration in white, but not grey, matter CBF in the same region has also been reported (Wijtenburg et al., 2017). These *in vivo* imaging studies are consistent with the expected direct relationship between glutamate and CBF via neurovascular coupling, but the paucity of evidence in schizophrenia underscores the need for more research. However, there is also evidence for disrupted neurovascular coupling in schizophrenia (Sukumar et al., 2020), which potentially could be more apparent in TRS.

Emerging evidence suggests that antipsychotic treatment dynamically affects the relationships between neurotransmitter activity and CBF. For example, risperidone treatment reversed the direction of the association between ACC Glx and the blood oxygen level-dependent (BOLD) response in schizophrenia after 6 weeks (Cadena et al., 2018). There is separate evidence that glutamate and CBF are modulated by antipsychotic treatment and that, potentially, this may contribute to symptomatic response. Longitudinal studies have suggested a pattern of decreasing glutamate levels during antipsychotic administration in schizophrenia, particularly in frontal regions (Egerton et al., 2017; Kubota et al., 2020). Decreases in glutamate following antipsychotic treatment are also supported by a recent meta-analysis, which shows a negative association between antipsychotic dose and glutamate or Glx concentration in the medial frontal cortex, including the ACC (Merritt et al., 2021). A separate meta-analysis indicated higher Glx in the dorsolateral prefrontal cortex and hippocampus only in unmedicated patients compared to healthy controls (Nakahara et al., 2022).

The efficacy of clozapine in TRS may also be partially related to glutamatergic effects, including positive modulation of NMDA receptor-mediated glutamate transmission (Arvanov et al., 1997; Arvanov and Wang, 1999; Chen and Yang, 2002; Gemperle et al., 2003; Gray et al., 2009; Homayoun and Moghaddam, 2007; Malhotra et al., 1997), suppression of hyperglutamatergic activity via activation of presynaptic group metabotropic glutamate receptors (e.g. III-mGluR) (Fukuyama et al., 2019), and attenuating MK-801-induced increases in glutamate release in the frontal cortex (López-Gil et al., 2007). We previously detected a decrease in striatal, but not ACC, glutamate levels during clozapine treatment in schizophrenia, which correlated with improvement in symptom severity (McQueen et al., 2021).

The changes in CBF observed during antipsychotic treatment appear to be regionally and potentially treatment-specific (Goozée et al., 2014). For example, haloperidol, a prototypical antipsychotic, decreased CBF in frontal and cerebellar regions and increased CBF in the basal ganglia (Lahti et al., 2003; Miller et al., 2001); while risperidone, an atypical antipsychotic, decreased CBF only in the cerebellar region (Miller et al., 2001). With regard to clozapine, mixed findings have been reported, including CBF decreases (Sun et al., 2024), increases (Ertugrul et al., 2009; Lahti et al., 2003; Sharafi, 2005), and no change in cortical regions (Zhao et al., 2006). Using the magnetic resonance imaging (MRI) approach of pseudo-continuous arterial spin labelling (pCASL), we recently reported decreases in CBF in the ACC over 12 weeks of clozapine treatment, consistent with some previous radiotracer studies (Lahti et al., 2003; Zhao et al., 2006), and that higher striatal and hippocampal CBF prior to commencing clozapine was associated with greater subsequent improvement in symptom severity (Sun et al., 2024).

The aim of this study was to examine the local relationships between glutamate metabolites and CBF in TRS and their change over 12 weeks of clozapine treatment. We hypothesised that, within the ACC and striatum, glutamate and CBF would be positively correlated, and that their changes over 12 weeks of clozapine treatment would also show a positive correlation. Exploratory analyses examined associations between glutamate, CBF, and symptom improvement. In addition, as glutamate may influence neural activity and thereby potentially CBF in distal brain regions (Duncan et al., 2014; Kiemes et al., 2021; Zahid et al., 2023), we also examined the relationships between ACC or striatal glutamate metabolites and CBF using a whole-brain voxel-wise approach.

## Methods

### Study design and participants

Ethical approval for this study was obtained from the London South East National Health Service Research Ethics Committee (Reference 13/LO/1857), and informed consent to participate was obtained from participants with the mental capacity to do so. For participants without the capacity to provide consent, a carer provided advice on their behalf.

Participants were recruited from inpatient and outpatient services at the South London and Maudsley and Oxleas NHS Foundation Trusts. Inclusion required that participants were ≥18 years old, met ICD-10 criteria for schizophrenia (F20) or schizoaffective disorder (F25), and were either clozapine naïve or had not taken clozapine for ≥3 months prior to the consent visit. Criteria for TRS were inferred from medical records and the treating psychiatrist, including having ≥2 previous trials of non-clozapine antipsychotics, each within the recommended dose for ≥6 weeks, as according to clinical judgement, and were being referred for clozapine initiation. Exclusion criteria for all participants included substance dependency as defined using ICD-10 criteria, pregnancy, or contraindication to MRI. The observation period was from baseline (after referral for clozapine but prior to clozapine initiation/reinitiation) to 12 weeks after clozapine initiation. The 12-week period was selected based on consensus guidelines, recommended for evaluating clozapine response (Howes et al., 2017).

### Clinical and demographic measures

Demographic variables included age and sex. Medical history was obtained through clinical interview and review of medical records and included age of onset (also used to calculate duration of illness), diagnosis, number of hospital admissions, and previous antipsychotic trials, name and dose of current antipsychotic prior to clozapine initiation, and any co-medications. At the time of each MRI visit, symptom severity was assessed using the Positive and Negative Syndrome Scale (PANSS; Kay et al., 1987), and social and occupational functioning was assessed with the Global Assessment of Functioning Scale (GAF) (Hall, 1995). Blood samples were taken to monitor clozapine plasma levels, to determine whether clozapine levels met the indicated therapeutic threshold of 350 ng/ml (Siskind et al., 2021) at week 6, week 12, and additionally at week 8 (if ≤350 ng/ml at week 6) (Rosenheck et al., 1999). Change in total symptom severity was calculated as the percentage change in PANSS total from baseline to week 12, calculated using the formula (Leucht et al., 2007):

$$\mathrm{Percent}\;\mathrm{Change}=[\frac{\left\{\mathrm{week}12-\mathrm{baseline}\right\}}{\left(\mathrm{baseline}-30\right)}]x100\%$$

## ^1^H-MRS Acquisition and Quantification

All data were acquired on a GE Healthcare MR750 3T scanner (Waukesha, USA) at the Centre for Neuroimaging Sciences, Institute of Psychiatry, Psychology & Neuroscience (King’s College London), across two sessions (‘baseline’, ‘week 12’). Acquisition of parameters for structural (T1-weighted) data was acquired using a 3D T1-weighted inversion recovery prepared spoiled gradient recalled sequence (Inversion time (TI) = 400 mseconds, Repetition time (TR) = 7.31 mseconds, Echo time (TE) = 3.02 mseconds, voxel size = .05 × 1.05 × 1.2 mm^3^, Field of view (FoV) = 270 mm, flip angle = 11°).

The proton magnetic resonance spectroscopy (^1^H-MRS) data was handled and described in the same manner, including parameters for data acquisition, analysis, and quality control, as in the study with an overlapping sample (McQueen et al., 2021). ^1^H-MRS glutamate and Glx levels were obtained from the conventional Point-Resolved Spectroscopy (PRESS) sequence, incorporating a CHEmically Selective Suppression (CHESS) water suppression routine (TE = 30 mseconds in the ACC and TE = 35 mseconds in the striatum, TR = 3000 mseconds, 96 averages). For each acquisition, unsuppressed water reference spectra (16 averages) were also acquired for eddy current correction and water scaling. The region of interest (ROI) was prescribed from the T1-weighted image, to be: (1) in the centre of the ACC (20 × 20 × 20 mm) (Egerton et al., 2012, 2018); (2) in the right striatum (20 × 20 × 20 mm, mainly covering the right head of striatum with the lower end of the voxel located dorsal to the anterior commissure, to include the maximum amount of grey matter and minimum amount of cerebrospinal fluid (CSF) (de la Fuente-Sandoval et al., 2013; McQueen et al., 2018). Images of voxel placement are provided in Figure S1.

Spectra were analysed using Linear Combination of Model Spectra (LCModel) version 6.3-0I (Provencher, 1993), using LCModel basis sets matched to the TEs for each voxel. Values for glutamate metabolites were corrected for voxel tissue composition by using the formula:

$$\begin{array}{l} \mathrm{Metabolite}\;\mathrm{Corrected}=\mathrm{Metabolite}\;\mathrm{Concentration}\\ \times \frac{\mathrm{WM}+1.21\mathrm{GM}+1.55\mathrm{CSF}}{\mathrm{WM}+\mathrm{GM}} \end{array}$$

where WM, GM, and CSF indicate the fraction of white and grey matter and cerebrospinal fluid content in the voxel. This formula assumes a CSF water concentration of 55,556 mol/m^3^ (Gasparovic et al., 2006). Voxel GM ratio was calculated as GM/(WM+GM) . Voxel WM, GM, and CSF content for each subject were determined by extracting the location of the voxel from the spectra file headers and using an in-house program to calculate the percentage of WM, GM, and CSF content using the segmented T1-weighted images. We used (1) visual inspection and (2) Cramér-Rao Lower Bound > 20% as reported by LCModel, which are estimates of the goodness of fit of the metabolite peaks, to exclude poorly fitted metabolite peaks from statistical analysis.

### pCASL Acquisition and Quantification

The data was handled and described in the same manner, including parameters for data acquisition, computation of CBF maps, and procedures for spatial normalisation of these maps to the Montreal Neurological Institute (MNI) reference space, as in the study with an overlapping sample (Sun et al., 2024). pCASL images were acquired using a 3D Fast Spin Echo spiral multi-shot readout. The parameters of the arterial labelling part of the sequence were: label duration = 1800 mseconds, post labelling delay = 2025 mseconds, TR = 4968 mseconds. For the readout part, 3D images were acquired with a Fast Spin Echo ‘stack of spirals’ routine, using 8 spiral arms per slice location, 512 points per arm, 55 slice locations of 3 mm thickness, TE = 11.09 mseconds, FoV = 240 mm. A proton-density image with the same parameters was collected within the same sequence, which was used to derive a whole brain CBF map in physiological units (ml blood/100 g tissue/minute) following the guidelines recently published for CBF computation (Alsop et al., 2015). The total acquisition time for the complete ASL pulse sequence and the proton-density image was in 6:28 minutes.

CBF maps were pre-processed and spatially normalised to the standard space of the MNI using Automatic Software for ASL Processing (ASAP) toolbox 26 (Mato Abad et al., 2016) running in Statistical Parametric Mapping Software (SPM-12, Functional Imaging Laboratory, Wellcome Centre for Human Neuroimaging, Institute of Neurology, University College London, UK, http://www.fil.ion.ucl.ac.uk/spm/software/spm12/). These spatially normalised images were used for ROI and voxel-wise analysis. For primary analyses, ROI masks in the bilateral ACC and striatum were defined using WFU PickAtlas in SPM-12 (‘ACC’ and ‘striatum’). For confirmatory analyses, as ^1^H-MRS data were acquired in the right striatum only, a separate ROI mask for the right striatum only was generated from the bilateral mask (anatomical ROI right striatum), and, to extract CBF values from the ^1^H-MRS voxel specifically, two additional masks were generated in SPM-12 from the ACC and right striatal ^1^H-MRS voxels. Mean CBF values were extracted from each mask using the ASAP toolbox. Mean global CBF values were extracted from the group grey matter mask to investigate global differences and to account for inter-individual differences in global perfusion.

### Statistical Analyses

#### Clinical data

The within-subject change in symptom severity and functioning following clozapine treatment was determined using paired t-tests with a two-tailed alpha of 0.05, in Python version 3.12.6.

#### Integration of ^1^H-MRS and pCASL data

##### ROI analysis

Shapiro-Wilk tests checked CBF and ^1^H-MRS variables for normality of distribution. Correlations between variables were analysed using Pearson’s tests. Associations between ACC or striatal glutamate, Glx and CBF within the same regions were determined at baseline, week 12, as well as their changes over 12 weeks. We gave a *p* value threshold of 0.05 and performed a Bonferroni correction for multiple comparisons over the two ROIs in two regions (*p* = 0.05/4 = 0.0125). For analyses relating to change over 12 weeks, potential influences of clozapine dose at follow-up were covaried for in secondary analyses.

Exploratory analyses of the relationships between glutamate concentrations, regional CBF, and symptoms were conducted using hierarchical linear regression. Separate hierarchical models examined the relationships between (a) change in glutamate (or Glx) and CBF and improvement in total symptom severity, and (b) the baseline glutamate (or Glx) and CBF and subsequent improvement in symptom severity. For analysis (a), change in global CBF was entered in step 1 (Model 1, control variable); change in glutamate metabolites and change in CBF values as predictors in step 2, covarying for change in global CBF (Model 2); and the interaction term change in glutamate metabolites × CBF values was entered in step 3 (Model 3). Similarly, for analysis (b), baseline global CBF was entered in step 1 (Model 1, control variable); baseline glutamate metabolites and baseline CBF values as predictors in step 2, controlling for baseline global CBF (Model 2); and the interaction term baseline glutamate metabolites x CBF values was entered in step 3 (Model 3). In addition, the week 12 clozapine dose was entered as a covariate (Model 4) in both analyses. The threshold for statistical significance was *p* < 0.05.

##### Whole-brain voxel-wise analyses

Supplementary voxel-wise analysis was conducted in SPM-12. To examine relationships between the change in glutamate (or Glx) and the change in CBF during clozapine treatment, using ImCalc, we first calculated one individual CBF difference image in MNI space for each participant (follow-up image – baseline image), representing the change in CBF during clozapine treatment. Changes in individual glutamate levels were then entered as regressors in a voxel-wise ANOVA, with or without week 12 clozapine dose entered as a covariate. To examine glutamate (or Glx)-CBF associations at the baseline and week 12 timepoints separately, individual participant glutamate concentration values were entered as regressors in a voxel-wise ANOVA, with or without global CBF as a covariate of no interest. The threshold for statistical significance was defined as *p* < 0.05, corrected for the Family-Wise Error (FWE) rate.

## Results

Both ^1^H-MRS and pCASL data were available for 30 individuals with TRS before clozapine initiation (baseline). Of these, data were available for 20 individuals at follow-up, 12 weeks after clozapine treatment. This study integrated a subsample of participants from previous publications reporting ^1^H-MRS (McQueen et al., 2021) and CBF (Sun et al., 2024) separately. Characteristics of the participants are shown in Table 1. Six of the 30 TRS subjects had previously taken clozapine. Symptom severity and functioning improved over 12 weeks of clozapine treatment. No significant demographic or clinical differences existed between those who did or did not complete the 12-week follow-up visit (Table S1). As previously reported (McQueen et al., 2021; Sun et al., 2024), ACC CBF (*t*(19) = 2.82, *p* = 0.01) and striatal glutamate (*t*(19) = 2.54, *p* = 0.02; Figure S2) decreased significantly with clozapine treatment (Table S2). The ^1^H-MRS-voxel-derived ACC CBF also showed a significant decrease (Table S2). These effects fell below the threshold for statistical significance when covarying for clozapine dose at 12 weeks. Values relating to ^1^H-MRS quality and voxel tissue composition at baseline and week 12 are available in Table S3.

**Table 1.** Descriptive statistics of the patient sample at baseline and follow-up.

Characteristic	Baseline		Week 12 Follow-up
	*N* = 30	*N* = 20 (with Follow-up)	*N* = 20
Age, years	37.83 (12.92)	38.25 (13.04)	
Sex, Male/Female	22/8	14/6	
Age of onset, years	24.73 (7.97)	26.00 (6.95)	
Duration of illness, years	13.93 (8.65)	13.35 (9.62)	
Diagnosis, F20-schizophrenia /F25-schizoaffective	25/5	17/3	
Number of hospital admissions, min; max; median; range	0; 12; 3; 12	0; 12; 3; 12	
Previous antipsychotic trials, min; max; median; range	2; 10; 3; 8	2; 7; 2.5; 5	
Previous clozapine use, Yes/No	6/24	2/18	
Antipsychotic prior to clozapine)			
Amisulpride	5	3	
Aripiprazole	3	2	
Flupentixol	2	2	
Haloperidol	2	1	
Olanzapine	7	4	
Quetiapine	4	4	
Risperidone	5	2	
Zuclopenthixol	1	1	
Paliperidone	1	1	
Antidepressant medication, N (Yes)	8	6	
GABAergic medication, N (Yes)	13	6	
Antipsychotic dose, CPZE/mg per day	238.93(192.74)	265.90 (220.99)	
Clozapine			
Clozapine dose, daily mg	/	/	336.3 (136.8)
Plasma clozapine, ng/ml	/	/	0.44 (0.29) ^†^
Plasma norclozapine, ng/ml	/	/	0.23 (0.12) ^‡^
Symptoms and functioning			
PANSS-Total	72.77 (15.58)	70.15 (16.30)	54.55 (13.75)
PANSS-Positive	19.17 (5.73)	18.35 (5.13)	13.80 (4.49)
PANSS-Negative	17.97 (6.50)	17.50 (6.80)	14.60 (5.54)
PANSS-General	35.63 (7.44)	34.30 (7.82)	26.55 (5.99)
GAF	46.10 (9.53)	49.60 (7.87)	60.20 (8.37)
Global CBF, ml/100 g/minute	40.07 (10.29)	38.74 (10.89)	36.14 (8.48)
Regional CBF, ml/100 g/minute			
ACC	38.33 (10.86)	37.40 (11.47)	33.20 (8.51)
Striatum	41.40 (7.85)	39.67 (7.61)	38.75 (7.54)
Glutamate			
ACC	14.27 (2.38)	14.45 (2.02)	14.61 (3.32)
Striatum	11.71 (1.65)	11.59 (1.68)	10.71 (1.53)
Glx			
ACC	19.92 (4.54)	20.38 (3.68)	20.74 (5.95)
Striatum	16.73 (3.43)	15.92 (3.57)	14.70 (2.95)

Data is presented as mean (standard deviation) unless otherwise specified.

ACC: Anterior cingulate cortex; CBF: Cerebral blood flow; CPZE: Chlorpromazine equivalent dose; GAF: Global assessment of functioning; Glx: Glutamate + Glutamine; GABA: γ-aminobutyric acid; GABAergic medication including benzodiazepines, valproate, lithium, zopiclone, pregabalin, lamotrigine; PANSS: Positive and Negative Syndrome Scale.

† *n* = 19.

‡ *n* = 18.

### Relationships between CBF and glutamate metabolites in the ACC and striatum

**Figure 1.**
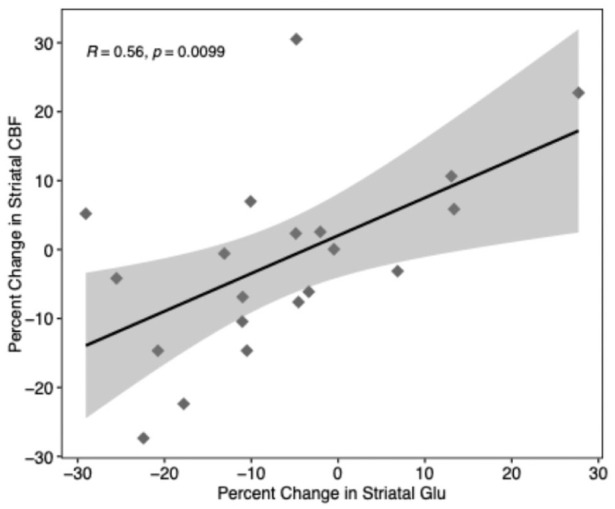
Scatterplot of change in glutamate and change in CBF during clozapine treatment in TRS (*N* = 20). ROI analysis showing correlations between change in striatal glutamate and change in striatal CBF. Scatterplots present individual values. The straight line is the line of best fit, with the 95% confidence interval indicated in grey. A positive percent change (%) indicates an increase in the value during treatment. CBF: Cerebral blood flow; Glu: Glutamate; ROI: Region of interest; TRS: Treatment-resistant schizophrenia.

At the baseline (prior to clozapine), there were no significant correlations between CBF and glutamate (or Glx) within the ACC or striatum (all *p* > 0.0125; Table 2). Over 12 weeks of clozapine treatment, a significant positive correlation was observed between changes in striatal CBF bilaterally and right striatal glutamate, such that reductions in CBF were associated with reductions in glutamate (*r* = 0.56, *p* < 0.01; 95% confidence intervals: 0.16, 0.80; Figure 1; Table 2). This effect remained significant after covarying for clozapine dose (*r* = 0.59, *p* = 0.01). This relationship was also significant when CBF was sampled from the anatomical ROI right striatum only (*r* = 0.56, *p* = 0.01), but fell below significance when sampling CBF from the right striatal ^1^H-MRS voxel (*r* = 0.38, *p* = 0.09). The positive relationship between changes in striatal CBF and striatal glutamate also remained significant (*r* = 0.50, *p* = 0.03) after exclusion of two potentially influential observations as identified using Cook’s D. No significant correlations were found between changes in glutamate metabolites and changes in CBF in the ACC; nor in either region at follow-up time points (all *p* > 0.0125; Table 2).

**Table 2.** Pearson’s correlations between CBF and glutamate metabolites in the ACC and striatum.

Change over 12 weeks						
Patients	CBF area	MRS metabolites	Pearson *r*	*N*	95%CI Lower, Upper	*p*
*N* = 20	ACC CBF	ACC Glu	0.02	20	−0.43, 0.46	0.93
		ACC Glx	−0.01	20	−0.45, 0.43	0.96
	Striatum CBF	Striatum Glu	0.56**	20	0.16, 0.80	**0.0099**
		Striatum Glx	0.38	20	−0.07, 0.71	0.09
Baseline (prior to clozapine initiation)						
*N* = 30	ACC CBF	ACC Glu	−0.12	30	−0.46, 0.25	0.54
		ACC Glx	−0.11	30	−0.45, 0.26	0.57
	Striatum CBF	Striatum Glu	0.09	30	−0.28, 0.43	0.65
		Striatum Glx	0.09	30	−0.28, 0.44	0.63
Follow-up (after 12 weeks of clozapine treatment)						
*N* = 20	ACC CBF	ACC Glutamate	−0.04	20	−0.48, 0.41	0.86
		ACC Glx	−0.49	20	−0.77, −0.06	0.03
	Striatum CBF	Striatum Glutamate	0.15	20	−0.31, 0.56	0.52
		Striatum Glx	0.34	20	−0.12, 0.68	0.14

ACC: Anterior cingulate cortex; CBF: Cerebral blood flow; Glu: Glutamate; Glx: Glutamate + Glutamine; MRS: Magnetic resonance spectroscopy.

** *p* < 0.01 (bolded values).

### Relationships with clinical variables

To investigate how CBF and glutamate metabolites may change in relation to clinical symptoms, we performed hierarchical multiple regression analyses. In these analyses, we first assessed regional CBF and glutamate or Glx in relation to total symptom improvement via multiple regression while considering the effect of global CBF. We then assessed the same relationship with interaction via moderation analyses. Finally, the clozapine dose at week 12 was included in an additional model.

The change over time in glutamate (or Glx) and CBF in the ACC and striatum did not significantly contribute to explaining variance in total symptom improvement during clozapine treatment (all *p* > 0.05; Table S4). However, when the week 12 clozapine dose was included, the clozapine dose significantly contributed to explaining variance in subsequent total symptom improvement (Table S5), while models examining change in CBF, glutamate, or Glx in the ACC in relation to symptom improvement became non-significant (all *p* > 0.05; Table S4; Table S5).

ACC CBF and ACC Glx prior to commencing clozapine significantly contributed to explaining variance in subsequent total symptom improvement during clozapine treatment (controlling for baseline global CBF: overall model: *R*^2^ = 0.41, *F* (3,16) = 3.72, *p* = 0.03), with this effect mainly driven by ACC Glx ( β =0.45, *p* = 0.03) rather than ACC CBF ( β = 0.97, *p* > 0.05); Table S6). The findings were similar when ACC CBF was sampled from the ^1^H-MRS voxel volume, with the overall model significantly contributing to total symptom improvement (controlling for baseline global CBF: overall model: *R*^2^ = 0.42, *F* (3,16) = 3.84, *p* = 0.03), again with this effect mainly driven by ACC Glx ( β =0.43, *p* = 0.04) rather than ACC CBF ( β = 0.58, *p* > 0.05). However, when the clozapine dose at 12 weeks was additionally included in the model, the relationships between baseline ACC CBF and Glx and subsequent symptom improvement fell below the threshold for significance (Table S7). The remaining models were non-significant (all *p* > 0.05).

### Voxel-wise analyses

**Figure 2.**
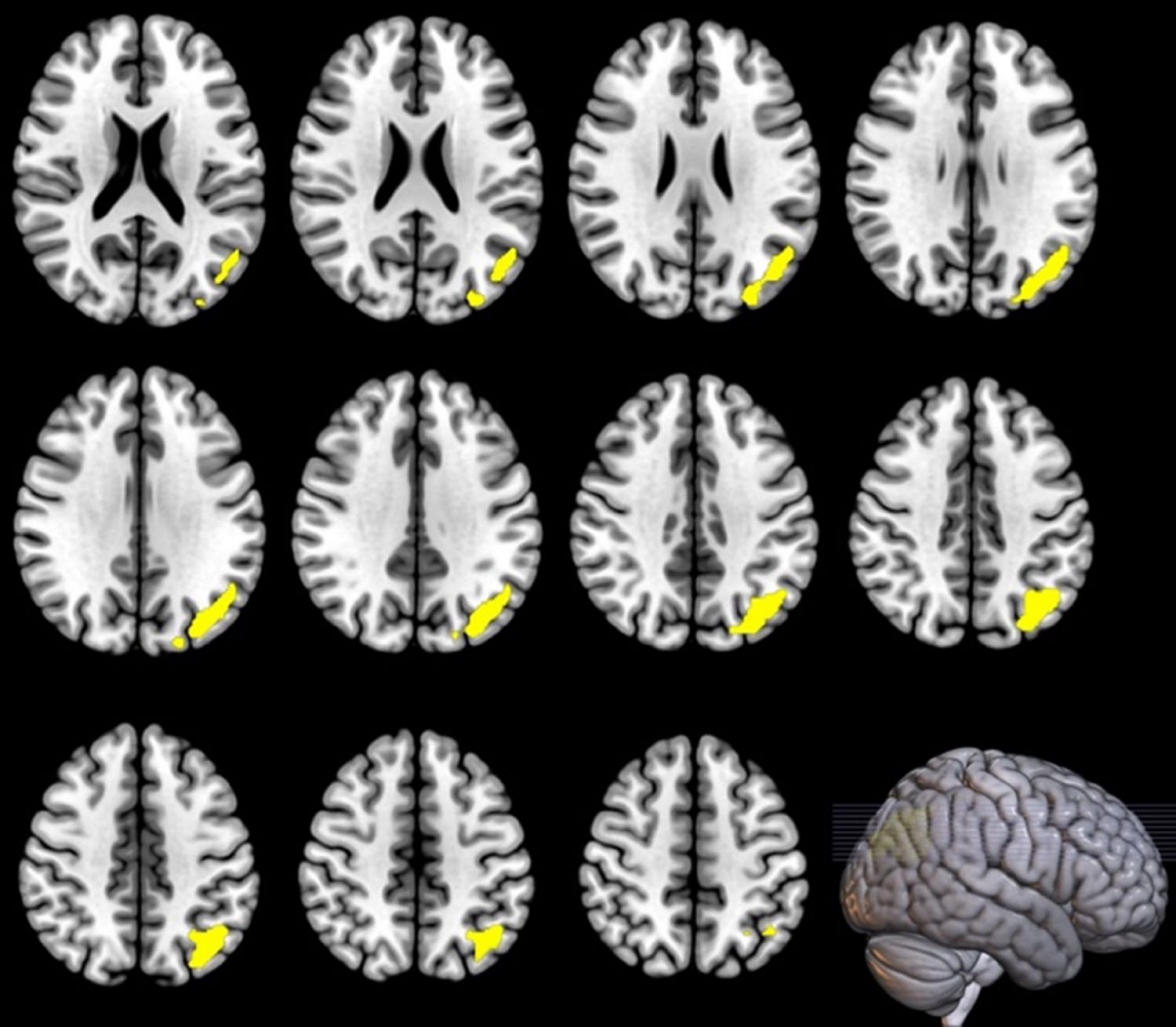
Relationship between change in striatal glutamate and change in cortical CBF during clozapine (*N* = 20). Highlighted brain sections illustrate the significant cluster (MNI (*x*, *y*, *z*) = −42, −66, 44; *p* < 0.05 family-wise error corrected) of positive relationships between change in CBF and change in glutamate. CBF: Cerebral blood flow; MNI: Montreal Neurological Institute.

**Figure 3.**
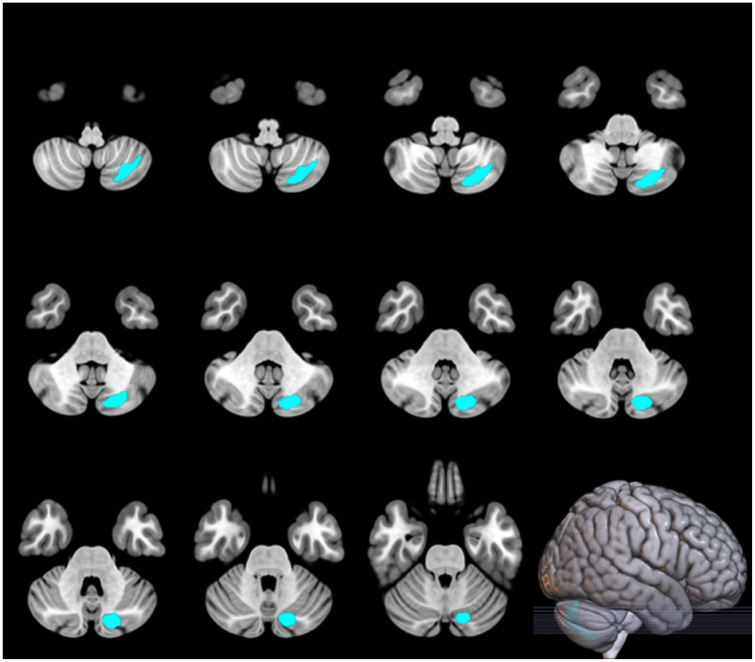
Relationship between ACC glutamate and cerebellar CBF after 12 weeks of clozapine (*N* = 20). Highlighted brain sections illustrate the significant cluster (MNI (*x*, *y*, *z*) = −18, −78, −38; *p* < 0.05 family-wise error corrected) of negative relationships between change in CBF and change in glutamate. CBF: Cerebral blood flow; MNI: Montreal Neurological Institute; ACC: Anterior cingulate cortex.

Voxel-wise analysis additionally detected a positive relationship between change in striatal glutamate and change in CBF in the parietal cortex during clozapine treatment (one cluster: MNI (*x*, *y*, *z*) = −42, −66, 44, *p* = 0.045 FWE; Figure 2), which remained significant when controlling for clozapine dose at week 12. There were no significant relationships between glutamate metabolites and CBF at baseline (all clusters *p* > 0.05 FWE). At the 12-week time-point, there was a negative relationship between ACC glutamate and CBF in the posterior lobe of the cerebellum covarying for global CBF (one cluster: MNI (*x*, *y*, *z*) = −18, −78, −38, *p* = 0.010 FWE; Figure 3).

## Discussion

This study examined the relationships between changes in brain glutamate and CBF over 12 weeks of clozapine treatment in TRS. Our main finding was that longitudinal changes in striatal glutamate and blood flow following clozapine treatment were positively correlated. Second, patients with higher Glx and CBF in the ACC prior to treatment tended to have better clinical improvement in total symptom severity during subsequent clozapine treatment, with this effect being significantly driven by ACC Glx, although these effects fell below the threshold for significance when the clozapine dose at week 12 was added to the model.

A positive association between reductions in striatal glutamate and striatal CBF during clozapine treatment is in keeping with a mechanistic association between neural glutamatergic activity and local CBF to support metabolism (Attwell et al., 2010), and indicates that reductions in striatal glutamate during clozapine treatment (McQueen et al., 2021) were correlated with functional consequences. According to established theories (Attwell et al., 2010), glutamate release increases intracellular Ca^2+^, which then triggers the release of vasoactive molecules, dilating blood vessel diameter (Filosa et al., 2004; Mishra et al., 2016; Zonta et al., 2003). Neurovascular coupling predicts that an increase in inhibitory activity will result in a decreased/negative BOLD functional MRI response (Donahue et al., 2010; Krause et al., 2014; Logothetis, 2008). Within our study, while it should be noted that ^1^H-MRS does not measure glutamate involved in neurotransmission specifically, the decrease in glutamate levels during clozapine treatment may indicate reduced excitatory activity, which could, in turn, lower metabolic demand and local CBF. Clozapine may act to normalise glutamatergic synaptic function (Hribkova et al., 2022), enhance NMDA receptor activity while blocking NMDA antagonist effects, and inhibit glutamate transmission (Fukuyama et al., 2019; Hribkova et al., 2022). Consequently, a decrease in striatal glutamate-mediated signalling may reduce neurovascular responses and decrease local blood flow (Shaw et al., 2021; Sun et al., 2013; Takano et al., 2006).

It is also possible that the observed positive relationship between the reduction in glutamate and the reduction in CBF in the striatum during clozapine treatment may reflect adaptive mechanisms related to discontinuing non-clozapine antipsychotics, either in addition to or instead of reflecting the effects of clozapine treatment specifically. For example, treatment with non-clozapine antipsychotics appears to be associated with increases in striatal blood flow (Goozée et al., 2014) and grey matter volume (Ebdrup et al., 2013; Jørgensen et al., 2016; Yang et al., 2021), whereas decreases in striatal volume are observed during clozapine treatment (Krajner et al., 2022; Sun et al., 2024). The relative contributions of non-clozapine antipsychotic discontinuation and clozapine treatment to striatal glutamate and CBF changes could be further explored in pre-clinical models. It is also possible that structural changes in the striatum could affect both striatal glutamate and CBF, although previous findings in this cohort suggest, changes in CBF and glutamate levels seem independent of the decrease in grey matter volume (Krajner et al., 2022; Sun et al., 2024).

In contrast to the striatum, although CBF was decreased in the ACC during clozapine treatment, this was not significantly associated with a change in ACC glutamate metabolites. This may indicate a lesser extent of neurovascular coupling in the ACC, and/or that the decrease in CBF in this region reflects non-glutamatergic processes, for example, changes in GABAergic activity, neuroinflammation, or vascular tone. Interestingly, a recent bimodal meta-analysis of regional CBF and glucose metabolism in schizophrenia indicates conjoint findings in the bilateral striatum, but disjoint findings, indicating neurovascular uncoupling, in dorsomedial frontal areas partially overlapping with the ACC region in our analysis (Sukumar et al., 2020).

Previous cross-sectional studies detected a positive relationship between glutamate and white matter CBF in individuals with schizophrenia (Wijtenburg et al., 2017) and between glutamate and grey matter CBF in healthy controls (Bojesen et al., 2018) in the ACC. We were unable to investigate the relationship between glutamate and white matter CBF due to the low CBF signal in white matter in our data. We did not observe any significant relationship between ACC glutamate and ACC grey matter CBF in participants with TRS prior to starting clozapine treatment, which may reflect illness or medication effects. Furthermore, ACC Glx predicted subsequent total symptom improvement during clozapine treatment, whereas ACC CBF did not contribute. This effect became non-significant when additional variance in symptom improvement was accounted for by including clozapine dose in the model. This exploratory finding warrants investigation in a larger, independent sample but may tentatively suggest that neurochemical signals can better predict clozapine response than blood flow.

The findings of this study should be considered in the context of its limitations, including the relatively small sample size of 20 participants at both time points. With 80% power, alpha level of 0.05, this sample size provides sensitivity to detect a correlation of approximately *r* = 0.55, uncorrected for multiple comparisons. For comparison, the observed association between changes in striatal CBF and striatal glutamate was of a similar effect size *r* = 0.56. The study may have been underpowered to detect associations of smaller magnitude. We did not examine glutamate-CBF relationships in patients with treatment-responsive illness, or their change during treatment with antipsychotics other than clozapine, or those resistant to clozapine. These comparisons would be required in future work to interpret findings in relation to TRS or clozapine treatment specifically. In addition, data in healthy control participants acquired using the same sequences over the same time period would have allowed us to interpret the patient findings in relation to measurement repeatability. At a field strength of 3 Tesla, the glutamate signal will also include contributions from glutamine, and glutamine cannot be accurately measured at 3 Tesla. ^1^H-MRS measures total MR-visible glutamate and glutamine in the voxel, rather than that involved in neurotransmission specifically. Excitatory and inhibitory neurotransmission involves glutamate-glutamine cycling and contributions from GABA, which was also not measured in this study. While our study applied ^1^H-MRS and pCASL to examine glutamate and CBF in the same participants, in future studies, this could be extended to examine relationships with GABA, metabolism (e.g. 18F-fluorodeoxyglucose positron emission tomography), functional connectivity, or markers of oxidative stress.

## Conclusions

In conclusion, our study provides evidence of a positive relationship between changes in glutamate and CBF in the striatum after switching from a non-clozapine antipsychotic to clozapine treatment. This indicates that glutamatergic changes, as observed with ^1^H-MRS, are accompanied by changes in brain activity. In contrast, this association was not observed in the ACC, which may reflect regional differences in neurovascular coupling in schizophrenia.

## Supplemental Material

sj-docx-1-jop-10.1177_02698811251409141 – Supplemental material for Associations between glutamate and cerebral blood flow in treatment-resistant schizophrenia during clozapine treatment Supplemental material, sj-docx-1-jop-10.1177_02698811251409141 for Associations between glutamate and cerebral blood flow in treatment-resistant schizophrenia during clozapine treatment by Junyu Sun, Fernando Zelaya, Kyra-Verena Sendt, Grant McQueen, Amy L. Gillespie, John Lally, Owen O’Daly, David J. Lythgoe, Oliver D. Howes, Gareth J. Barker, Philip McGuire, James H. MacCabe and Alice Egerton in Journal of Psychopharmacology
