# Supplementary material for: Associations between glutamate and cerebral blood flow in treatment-resistant schizophrenia during clozapine treatment
Source: J Psychopharmacol. 2026 Jan 8;40(4):563–73. doi: 10.1177/02698811251409141 (PMC13283506; doi:10.1177/02698811251409141)
Supplement: sj-docx-1-jop-10.1177_02698811251409141 – Supplemental material for Associations between glutamate and cerebral blood flow in treatment-resistant schizophrenia during clozapine treatment [file sj-docx-1-jop-10.1177_02698811251409141.docx]

Supplementary material

**Associations between glutamate and cerebral blood flow in treatment resistant schizophrenia during clozapine treatment.**

Junyu Sun, Fernando Zelaya, Kyra-Verena Sendt, Grant McQueen, Amy L. Gillespie, John Lally, Owen O'Daly, David J. Lythgoe, Oliver D. Howes, Gareth J. Barker, Philip McGuire, James H. MacCabe and Alice Egerton.

Table of Contents

[Supplementary Tables 2](#_Toc213679426)

[Table S1. Baseline demographic and clinical characteristics of patients who did (N=20) or did not (N=10) complete follow-up MRI at week 12. 2](#_Toc213679427)

[Table S2. Statistical comparisons at baseline vs. week 12 (N=20). 3](#_Toc213679428)

[Table S3. Indices of spectral quality and tissue-fraction characteristics in the anterior cingulate cortex and striatum at baseline and week 12, in the total sample and those who completed follow-up scans at week 12. 4](#_Toc213679429)

[Table S4. Hierarchical regression of associations between change in CBF and glutamate metabolites in the ACC and striatum during clozapine treatment and change in PANSS total scores (N=20). 5](#_Toc213679430)

[Table S5. Hierarchical regression of associations between change in CBF and glutamate metabolites in the ACC and striatum during clozapine treatment and change in PANSS total scores, including clozapine dose at week 12 (N=20). 7](#_Toc213679431)

[Table S6. Hierarchical regression of associations between baseline CBF and glutamate metabolites in the ACC and striatum prior to clozapine treatment and change in PANSS total scores (N=20). 9](#_Toc213679432)

[Table S7. Hierarchical regression of associations between baseline CBF and glutamate metabolites in the ACC and striatum prior to clozapine treatment and change in PANSS total scores, including clozapine dose at week 12 (N=20). 11](#_Toc213679433)

[Supplementary Figures 13](#_Toc213679434)

[Figure S1. Representative images of ^1^H-MRS voxel placement the anterior cingulate cortex (A) and striatum (B). 13](#_Toc213679435)

[Figure S2. Striatal glutamate before and after 12 weeks of clozapine treatment (N=20). 13](#_Toc213679436)

# Supplementary Tables

## Table S1. Baseline demographic and clinical characteristics of patients who did (N=20) or did not (N=10) complete follow-up MRI at week 12.

| **Baseline** | **Week 12 Completers** | **Week 12**  **Non-completers** | $\boldsymbol{t/}\boldsymbol{F}$ | $\boldsymbol{P}$ |
| --- | --- | --- | --- | --- |
|  | **N=20** | **N=10** |  |  |
| Age, years | 38.25 (13.04) | 37.00 (13.32) | 0.25 | 0.81 |
| Sex, Male/Female | 14/6 | 8/2 | 0.34 | 0.45 |
| Age of onset, years | 26.00 (6.95) | 22.20 (9.60) | 1.24 | 0.22 |
| Duration of illness, years | 13.35 (9.62) | 15.10 (6.57) | -0.52 | 0.61 |
| Previous clozapine use, Yes/No | 2/18 | 4/6 | 3.75 | 0.08 |
| Diagnosis, F20-schizophrenia/F25-schizoaffective | 17/3 | 8/2 | 0.12 | 0.55 |
| Antipsychotic dose, CPZE/mg per day | 265.9 (221.0) | 185.0 (108.4) | 1.09 | 0.29 |
| Global CBF, ml/100g/min | 38.74 (10.89) | 42.75 (8.89) | -1.01 | 0.32 |
| Regional CBF, ml/100g/min | | | | |
| ACC | 37.40 (11.47) | 40.19 (9.80) | -0.66 | 0.52 |
| Striatum | 39.67 (7.61) | 44.86 (7.49) | -1.77 | 0.09 |
| Glutamate |  |  |  |  |
| ACC | 14.45 (2.02) | 13.91 (3.06) | 0.58 | 0.57 |
| Striatum | 11.59 (1.68) | 11.94 (1.66) | -0.53 | 0.60 |
| Glx |  |  |  |  |
| ACC | 20.38 (3.68) | 19.00 (6.05) | 0.78 | 0.44 |
| Striatum | 15.92 (3.57) | 18.35 (2.59) | -1.91 | 0.07 |
| Previous antipsychotic trials,  min; max; median; range | 2; 7; 2.5; 5 | 2; 10; 5; 8 | 9.27 | 0.16 |
| Number of hospital admissions,  min; max; median; range | 0; 12; 3; 12 | 1; 12; 5; 11 | 9.15 | 0.33 |
| Symptoms and Functioning |  |  |  |  |
| PANSS-Positive | 18.35 (5.13) | 20.80 (6.76) | -1.11 | 0.28 |
| PANSS-Negative | 17.50 (6.80) | 18.90 (6.08) | -0.55 | 0.59 |
| PANSS-General | 34.30 (7.82) | 38.30 (6.11) | -1.41 | 0.17 |
| PANSS-Total | 70.15 (16.30) | 78.00 (13.25) | -1.32 | 0.20 |

Data is presented as mean (standard deviation) unless otherwise specified. ACC: anterior cingulate cortex; CBF: cerebral blood flow; CPZE: chlorpromazine equivalent dose; Glx: glutamate + glutamine; PANSS: positive and negative syndrome scale.

## Table S2. Statistical comparisons at baseline vs. week 12 (N=20).

| **N=20** | **Baseline** | **Week 12** | $\boldsymbol{t}$ | $\boldsymbol{df}$ | $\boldsymbol{P}$ |
| --- | --- | --- | --- | --- | --- |
| Symptoms and Functioning |  |  |  |  |  |
| PANSS-Total | 70.15 (16.30) | 54.55 (13.75) | 6.87 | 19 | < 0.01** |
| PANSS-Positive | 18.35 (5.13) | 13.80 (4.49) | 7.47 | 19 | < 0.01** |
| PANSS-Negative | 17.50 (6.80) | 14.60 (5.54) | 2.67 | 19 | 0.02* |
| PANSS-General | 34.30 (7.82) | 26.55 (5.99) | 5.58 | 19 | < 0.01** |
| GAF | 49.60 (7.87) | 60.20 (8.37) | -4.84 | 19 | < 0.01** |
| Global CBF, ml/100g/min | 38.74 (10.89) | 36.14 (8.48) | 1.70 | 19 | 0.11 |
| Regional CBF, ml/100g/min | | | | | |
| ACC | 37.40 (11.47) | 33.20 (8.51) | 2.82 | 19 | 0.01* |
| MRS ACC | 50.88 (14.53) | 44.32 (10.20) | 3.16 | 19 | 0.01* |
| Striatum | 39.67 (7.61) | 38.75 (7.54) | 0.74 | 19 | 0.47 |
| Right Striatum | 39.55 (7.58) | 39.27 (8.61) | 0.22 | 19 | 0.83 |
| MRS Striatum | 38.85 (8.71) | 37.95 (7.63) | 0.50 | 19 | 0.62 |
| Glutamate |  |  |  |  |  |
| ACC | 14.45 (2.02) | 14.61 (3.32) | -0.22 | 19 | 0.83 |
| Striatum | 11.59 (1.68) | 10.71 (1.53) | 2.54 | 19 | 0.02* |
| Glx |  |  |  |  |  |
| ACC | 20.38 (3.68) | 20.74 (5.95) | -0.29 | 19 | 0.78 |
| Striatum | 15.92 (3.57) | 14.70 (2.95) | 1.63 | 19 | 0.12 |

Data are presented as Mean (Standard Deviation). ACC, Anterior Cingulate Cortex; CBF, Cerebral Blood Flow; Glx, Glutamate + Glutamine; GAF, Global Assessment of Functioning; PANSS, Positive and Negative Syndrome Scale; Regional CBF values extracted from the Magnetic Resonance Spectroscopy (MRS) voxel are labelled MRS ACC and MRS striatum.

* $\boldsymbol{P}<0.05$

** $\boldsymbol{P}<0.01$

## Table S3. Indices of spectral quality and tissue-fraction characteristics in the anterior cingulate cortex and striatum at baseline and week 12, in the total sample and those who completed follow-up scans at week 12.

|  | **Baseline**  **total sample**  **N=30** | **Baseline Completers**  **N=20** | **Week 12**  **N=20** | $\boldsymbol{P}$ |
| --- | --- | --- | --- | --- |
| ACC |  |  |  |  |
| SNR | 23.17 (3.97) | 23.00 (3.57) | 21.60 (4.96) | 0.17 |
| Linewidth, at FWHM | 0.04 (0.01) | 0.04 (0.01) | 0.06 (0.08) | 0.23 |
| Voxel GM | 0.58 (0.09) | 0.60 (0.09) | 0.56 (0.06) | 0.01* |
| Voxel WM | 0.13 (0.05) | 0.11 (0.04) | 0.13 (0.05) | 0.11 |
| Voxel CSF | 0.29 (0.11) | 0.28 (0.11) | 0.31 (0.08) | 0.14 |
| Striatum |  |  |  |  |
| SNR | 17.57 (3.81) | 18.45 (3.49) | 17.50 (3.94) | 0.30 |
| Linewidth, at FWHM | 0.07 (0.01) | 0.07 (0.01) | 0.08 (0.02) | 0.13 |
| Voxel GM | 0.50 (0.06) | 0.51 (0.07) | 0.52 (0.07) | 0.66 |
| Voxel WM | 0.46 (0.07) | 0.47 (0.07) | 0.45 (0.08) | 0.35 |
| Voxel CSF | 0.04 (0.04) | 0.02 (0.02) | 0.02 (0.03) | 0.58 |

Data shown as mean (Standard Deviation). The P values relate to the significance of paired samples t-tests of data at baseline and week 12 in participants who completed both visits (N=20). ACC: Anterior Cingulate Cortex; CSF: Cerebral Spinal Fluid; GM: Grey Matter; Linewidth, at FWHM: Full Width at Half Maximum; SNR: Signal to Noise Ratio; WM: White Matter. * $\boldsymbol{P}<0.05$

## Table S4. Hierarchical regression of associations between change in CBF and glutamate metabolites in the ACC and striatum during clozapine treatment and change in PANSS total scores (N=20).

|  | | | | | | | | | |
| --- | --- | --- | --- | --- | --- | --- | --- | --- | --- |
| **ACC** | **Model 1** | | | **Model 2** | | | **Model 3** | | |
|  |  | | |  | | |  | | |
|  |  | | |  | | |  | | |
| **Step 1: Covariates** | $\boldsymbol{B(\beta)}$ | $\boldsymbol{t}$ | $\boldsymbol{P}$ | $\boldsymbol{B(\beta)}$ | $\boldsymbol{t}$ | $\boldsymbol{P}$ | $\boldsymbol{B(\beta)}$ | $\boldsymbol{t}$ | $\boldsymbol{P}$ |
| Change in global CBF | 0.54 (0.35) | 1.60 | 0.13 | 1.09 (0.70) | 1.76 | 0.10 | 1.20 (0.77) | 1.95 | 0.07 |
| **Step 2: Predictors** |  |  |  |  |  |  |  |  |  |
| Change in ACC CBF |  |  |  | -0.66 (-0.46) | -1.15 | 0.27 | -0.81 (-0.56) | -1.41 | 0.18 |
| Change in ACC Glutamate |  |  |  | -0.30 (-0.31) | -1.43 | 0.17 | -0.04 (-0.04) | -0.12 | 0.91 |
| **Step 3: Interaction terms** |  |  |  |  |  |  |  |  |  |
| Change in ACC CBF x Change in ACC Glutamate |  |  |  |  |  |  | 0.02 (0.37) | 1.24 | 0.23 |
| $\boldsymbol{R}$**^2^** | 0.12 | | | 0.31 | | | 0.37 | | |
| $\boldsymbol{\Delta R}$**^2^** |  | | | 0.19 | | | 0.06 | | |
| **F-statistic (df_1_, df_2_)** | 2.55 (1, 18) | | | 2.38 (3, 16) | | | 2.23 (4, 15) | | |
|  |  |  |  |  |  |  |  |  |  |
|  |  |  |  |  |  |  |  |  |  |
| **Step 1: Covariates** | $\boldsymbol{B(\beta)}$ | $\boldsymbol{t}$ | $\boldsymbol{P}$ | $\boldsymbol{B(\beta)}$ | $\boldsymbol{t}$ | $\boldsymbol{P}$ | $\boldsymbol{B(\beta)}$ | $\boldsymbol{t}$ | $\boldsymbol{P}$ |
| Change in global CBF | 0.54 (0.35) | 1.60 | 0.13 | 1.35 (0.87) | 2.15 | 0.047* | 1.36 (0.88) | 2.03 | 0.06 |
| **Step 2: Predictors** |  |  |  |  |  |  |  |  |  |
| Change in ACC CBF |  |  |  | -0.87 (-0.61) | -1.50 | 0.15 | -0.88 (-0.61) | -1.42 | 0.18 |
| Change in ACC Glx |  |  |  | -0.13 (-0.16) | -0.73 | 0.47 | -0.12 (-0.15) | -0.46 | 0.65 |
| **Step 3: Interaction terms** |  |  |  |  |  |  |  |  |  |
| Change in ACC CBF x Change in ACC Glx |  |  |  |  |  |  | 0.00 (0.01) | 0.04 | 0.97 |
| $\boldsymbol{R}$**^2^** | 0.12 | | | 0.25 | | | 0.25 | | |
| $\boldsymbol{\Delta R}$**^2^** |  | | | 0.13 | | | 0.00 | | |
| **F-statistic (df_1_, df_2_)** | 2.55 (1, 18) | | | 1.74 (3, 16) | | | 1.23 (4, 15) | | |
|  |  |  |  |  |  |  |  |  |  |
|  |  |  |  |  |  |  |  |  |  |
| **Striatum** | **Model 1** | | | **Model 2** | | | **Model 3** | | |
|  |  |  |  |  |  |  |  |  |  |
|  |  |  |  |  |  |  |  |  |  |
| **Step 1: Covariates** | $\boldsymbol{B(\beta)}$ | $\boldsymbol{t}$ | $\boldsymbol{P}$ | $\boldsymbol{B(\beta)}$ | $\boldsymbol{t}$ | $\boldsymbol{P}$ | $\boldsymbol{B(\beta)}$ | $\boldsymbol{t}$ | $\boldsymbol{P}$ |
| Change in global CBF | 0.54 (0.35) | 1.60 | 0.13 | 0.96 (0.62) | 2.23 | 0.04* | 1.17 (0.76) | 2.42 | 0.03* |
| **Step 2: Predictors** |  |  |  |  |  |  |  |  |  |
| Change in Striatum CBF |  |  |  | -1.11 (-0.65) | -2.20 | 0.04* | -0.96 (-0.57) | -1.84 | 0.09 |
| Change in Striatum Glutamate |  |  |  | 0.59 (0.36) | 1.44 | 0.17 | 0.36 (0.21) | 0.73 | 0.47 |
| **Step 3: Interaction terms** |  |  |  |  |  |  |  |  |  |
| Change in Striatum CBF x Change in Striatum Glutamate |  |  |  |  |  |  | 0.03 (0.26) | 0.96 | 0.35 |
| $\boldsymbol{R}$**^2^** | 0.12 | | | 0.34 | | | 0.38 | | |
| $\boldsymbol{\Delta R}$**^2^** |  | | | 0.22 | | | 0.04 | | |
| **F-statistic (df_1_, df_2_)** | 2.55 (1, 18) | | | 2.77 (3, 16) | | | 2.30 (4, 15) | | |
|  |  |  |  |  |  |  |  |  |  |
|  |  |  |  |  |  |  |  |  |  |
| **Step 1: Covariates** | $\boldsymbol{B(\beta)}$ | $\boldsymbol{t}$ | $\boldsymbol{P}$ | $\boldsymbol{B(\beta)}$ | $\boldsymbol{t}$ | $\boldsymbol{P}$ | $\boldsymbol{B(\beta)}$ | $\boldsymbol{t}$ | $\boldsymbol{P}$ |
| Change in global CBF | 0.54 (0.35) | 1.60 | 0.13 | 1.05 (0.68) | 2.33 | 0.03* | 1.22 (0.79) | 2.43 | 0.03* |
| **Step 2: Predictors** |  |  |  |  |  |  |  |  |  |
| Change in Striatum CBF |  |  |  | -0.86 (-0.50) | -1.67 | 0.11 | -0.88 (-0.52) | -1.70 | 0.11 |
| Change in Striatum Glx |  |  |  | 0.04 (0.04) | 0.15 | 0.88 | 0.15 (0.13) | 0.48 | 0.64 |
| **Step 3: Interaction terms** |  |  |  |  |  |  |  |  |  |
| Change in Striatum CBF x Change in Striatum Glx |  |  |  |  |  |  | 0.02 (0.23) | 0.80 | 0.44 |
| $\boldsymbol{R}$**^2^** | 0.12 | | | 0.26 | | | 0.29 | | |
| $\boldsymbol{\Delta R}$**^2^** |  | | | 0.14 | | | 0.03 | | |
| **F statistic (df_1_, df_2_)** | 2.55 (1, 18) | | | 1.85 (3, 16) | | | 1.52 (2, 15) | | |
|  |  |  |  |  |  |  |  |  |  |

Change in global CBF was entered in step 1 (Model 1, control variable); Change in glutamate metabolites and change in CBF values as predictors in step 2, covarying for change in global CBF (Model 2); The interaction term change in glutamate metabolites x CBF values was entered in step 3 (Model 3).

$B$: Unstandardized coefficient; $\beta$: standardized beta coefficient; $R$^2^: proportion of variance in dependent variable explained by the model; $\Delta R$^2^: change in $R$^2^ from the previous step; df_1_: numerator degrees of freedom; df_2_: denominator degrees of freedom; F statistic: F-test of model. ACC: Anterior Cingulate Cortex; CBF: Cerebral Blood Flow; Glx: Glutamate + Glutamine. * $\boldsymbol{P}<0.05$

##

## Table S5. Hierarchical regression of associations between change in CBF and glutamate metabolites in the ACC and striatum during clozapine treatment and change in PANSS total scores, including clozapine dose at week 12 (N=20).

|  | | | | | | | | | | | | |
| --- | --- | --- | --- | --- | --- | --- | --- | --- | --- | --- | --- | --- |
| **ACC** | **Model 1** | | | **Model 2** | | | **Model 3** | | | **Model 4** | | |
|  |  | | |  | | |  | | |  | | |
|  |  | | |  | | |  | | |  | | |
| **Step 1: Covariates** | $\boldsymbol{B(\beta)}$ | $\boldsymbol{t}$ | $\boldsymbol{P}$ | $\boldsymbol{B(\beta)}$ | $\boldsymbol{t}$ | $\boldsymbol{P}$ | $\boldsymbol{B(\beta)}$ | $\boldsymbol{t}$ | $\boldsymbol{P}$ | $\boldsymbol{B(\beta)}$ | $\boldsymbol{t}$ | $\boldsymbol{P}$ |
| Change in global CBF | 0.51 (0.35) | 1.53 | 0.14 | 0.94 (0.64) | 1.54 | 0.14 | 1.05 (0.71) | 1.73 | 0.11 | 0.93 (0.63) | 1.72 | 0.11 |
| **Step 2: Predictors** |  |  |  |  |  |  |  |  |  |  |  |  |
| Change in ACC CBF |  |  |  | -0.53 (-0.39) | -0.95 | 0.36 | -0.68 (-0.50) | -1.20 | 0.25 | -0.54 (-0.40) | -1.08 | 0.30 |
| Change in ACC Glutamate |  |  |  | -0.33 (-0.35) | -1.60 | 0.13 | -0.08 (-0.09) | -0.28 | 0.78 | -0.08 (-0.09) | -0.31 | 0.76 |
| **Step 3: Interaction terms** |  |  |  |  |  |  |  |  |  |  |  |  |
| Change in ACC CBF x Change in ACC Glutamate |  |  |  |  |  |  | 0.02 (0.36) | 1.19 | 0.25 | 0.01 (0.20) | 0.71 | 0.49 |
| **Step 4: Clozapine dose** |  |  |  |  |  |  |  |  |  |  |  |  |
| Week 12 clozapine dose |  |  |  |  |  |  |  |  |  | 0.07 (0.45) | 2.22 | 0.04* |
| $\boldsymbol{R}$**^2^** | 0.12 | | | 0.32 | | | 0.38 | | | 0.55 | | |
| $\boldsymbol{\Delta R}$**^2^** |  | | | 0.20 | | | 0.06 | | | 0.17 | | |
| **F-statistic (df_1_, df_2_)** | 2.35 (1, 17) | | | 2.36 (3, 15) | | | 2.17 (4, 14) | | | 3.22 (5, 13) * | | |
|  |  |  |  |  |  |  |  |  |  |  |  |  |
|  |  |  |  |  |  |  |  |  |  |  |  |  |
| **Step 1: Covariates** | $\boldsymbol{B(\beta)}$ | $\boldsymbol{t}$ | $\boldsymbol{P}$ | $\boldsymbol{B(\beta)}$ | $\boldsymbol{t}$ | $\boldsymbol{P}$ | $\boldsymbol{B(\beta)}$ | $\boldsymbol{t}$ | $\boldsymbol{P}$ | $\boldsymbol{B(\beta)}$ | $\boldsymbol{t}$ | $\boldsymbol{P}$ |
| Change in global CBF | 0.51 (0.35) | 1.53 | 0.14 | 1.23 (0.84) | 1.97 | 0.07 | 1.23 (0.84) | 1.84 | 0.09 | 0.96 (0.65) | 1.67 | 0.12 |
| **Step 2: Predictors** |  |  |  |  |  |  |  |  |  |  |  |  |
| Change in ACC CBF |  |  |  | -0.78 (-0.57) | -1.34 | 0.20 | -0.77 (-0.57) | -1.25 | 0.23 | -0.54 (-0.40) | -1.02 | 0.33 |
| Change in ACC Glx |  |  |  | -0.14 (-0.18) | -0.80 | 0.43 | -0.14 (-0.19) | -0.55 | 0.59 | -0.05 (-0.07) | -0.24 | 0.82 |
| **Step 3: Interaction terms** |  |  |  |  |  |  |  |  |  |  |  |  |
| Change in ACC CBF x Change in ACC Glx |  |  |  |  |  |  | -0.00 (-0.01) | -0.03 | 0.97 | -0.00 (-0.08) | -0.26 | 0.80 |
| **Step 4: Clozapine dose** |  |  |  |  |  |  |  |  |  |  |  |  |
| Week 12 clozapine dose |  |  |  |  |  |  |  |  |  | 0.09 (0.54) | 2.59 | 0.02 |
| $\boldsymbol{R}$**^2^** | 0.12 | | | 0.24 | | | 0.24 | | | 0.50 | | |
| $\boldsymbol{\Delta R}$**^2^** |  | | | 0.12 | | | 0.00 | | | 0.26 | | |
| **F-statistic (df_1_, df_2_)** | 2.35 (1, 17) | | | 1.55 (3, 15) | | | 1.09 (4, 14) | | | 2.56 (5, 13) | | |
|  |  |  |  |  |  |  |  |  |  |  |  |  |
|  |  |  |  |  |  |  |  |  |  |  |  |  |
| **Striatum** | **Model 1** | | | **Model 2** | | | **Model 3** | | | **Model 4** | | |
|  |  |  |  |  |  |  |  |  |  |  |  |  |
|  |  |  |  |  |  |  |  |  |  |  |  |  |
| **Step 1: Covariates** | $\boldsymbol{B(\beta)}$ | $\boldsymbol{t}$ | $\boldsymbol{P}$ | $\boldsymbol{B(\beta)}$ | $\boldsymbol{t}$ | $\boldsymbol{P}$ | $\boldsymbol{B(\beta)}$ | $\boldsymbol{t}$ | $\boldsymbol{P}$ | $\boldsymbol{B(\beta)}$ | $\boldsymbol{t}$ | $\boldsymbol{P}$ |
| Change in global CBF | 0.51 (0.35) | 1.53 | 0.14 | 0.85 (0.58) | 2.19 | 0.04* | 0.89 (0.60) | 1.84 | 0.09 | 0.93 (0.63) | 2.28 | 0.04* |
| **Step 2: Predictors** |  |  |  |  |  |  |  |  |  |  |  |  |
| Change in Striatum CBF |  |  |  | -1.19 (-0.74) | -2.62 | 0.02* | -1.17 (-0.73) | -2.34 | 0.03* | -0.59 (-0.37) | -1.22 | 0.25 |
| Change in Striatum Glutamate |  |  |  | 0.85 (0.53) | 2.17 | 0.04* | 0.81 (0.50) | 1.57 | 0.14 | 0.29 (0.18) | 0.59 | 0.56 |
| **Step 3: Interaction terms** |  |  |  |  |  |  |  |  |  |  |  |  |
| Change in Striatum CBF x Change in Striatum Glutamate |  |  |  |  |  |  | 0.00 (0.03) | 0.12 | 0.91 | 0.03 (0.30) | 1.11 | 0.29 |
| **Step 4: Clozapine dose** |  |  |  |  |  |  |  |  |  |  |  |  |
| Week 12 clozapine dose |  |  |  |  |  |  |  |  |  | 0.08 (0.51) | 2.55 | 0.02* |
| $\boldsymbol{R}$**^2^** | 0.12 | | | 0.44 | | | 0.44 | | | 0.63 | | |
| $\boldsymbol{\Delta R}$**^2^** |  | | | 0.32 | | | 0.00 | | | 0.19 | | |
| **F-statistic (df_1_, df_2_)** | 2.35 (1, 17) | | | 3.91 (3, 15) * | | | 2.74 (4, 14) | | | 4.36 (5, 13) * | | |
|  |  |  |  |  |  |  |  |  |  |  |  |  |
|  |  |  |  |  |  |  |  |  |  |  |  |  |
| **Step 1: Covariates** | $\boldsymbol{B(\beta)}$ | $\boldsymbol{t}$ | $\boldsymbol{P}$ | $\boldsymbol{B(\beta)}$ | $\boldsymbol{t}$ | $\boldsymbol{P}$ | $\boldsymbol{B(\beta)}$ | $\boldsymbol{t}$ | $\boldsymbol{P}$ | $\boldsymbol{B(\beta)}$ | $\boldsymbol{t}$ | $\boldsymbol{P}$ |
| Change in global CBF | 0.51 (0.35) | 1.53 | 0.14 | 0.97 (0.66) | 2.25 | 0.04* | 1.13 (0.77) | 2.36 | 0.03* | 0.97 (0.66) | 2.50 | 0.03* |
| **Step 2: Predictors** |  |  |  |  |  |  |  |  |  |  |  |  |
| Change in Striatum CBF |  |  |  | -0.95 (-0.59) | -1.94 | 0.07 | -0.97 (-0.60) | -1.96 | 0.07 | -0.63 (-0.39) | -1.51 | 0.15 |
| Change in Striatum Glx |  |  |  | 0.29 (0.23) | 0.95 | 0.36 | 0.39 (0.31) | 1.17 | 0.26 | 0.43 (0.35) | 1.63 | 0.13 |
| **Step 3: Interaction terms** |  |  |  |  |  |  |  |  |  |  |  |  |
| Change in Striatum CBF x Change in Striatum Glx |  |  |  |  |  |  | 0.02 (0.23) | 0.81 | 0.43 | 0.03 (0.36) | 1.55 | 0.15 |
| **Step 4: Clozapine dose** |  |  |  |  |  |  |  |  |  |  |  |  |
| Week 12 clozapine dose |  |  |  |  |  |  |  |  |  | 0.09 (0.55) | 2.97 | 0.01* |
| $\boldsymbol{R}$**^2^** | 0.12 | | | 0.30 | | | 0.34 | | | 0.60 | | |
| $\boldsymbol{\Delta R}$**^2^** |  | | | 0.18 | | | 0.04 | | | 0.26 | | |
| **F statistic (df_1_, df_2_)** | 2.35 (1, 17) | | | 2.19 (3, 15) | | | 1.76 (4, 14) | | | 3.96 (5, 13) * | | |
|  |  |  |  |  |  |  |  |  |  |  |  |  |

Change in global CBF was entered in step 1 (Model 1, control variable); Change in glutamate metabolites and change in CBF values as predictors in step 2, covarying for change in global CBF (Model 2); The interaction term change in glutamate metabolites x CBF values was entered in step 3 (Model 3); Week 12 clozapine dose was entered as covariates (Model 4).

$B$: Unstandardized coefficient; $\beta$: standardized beta coefficient; $R$^2^: proportion of variance in dependent variable explained by the model; $\Delta R$^2^: change in $R$^2^ from the previous step; df_1_: numerator degrees of freedom; df_2_: denominator degrees of freedom; F statistic: F-test of model. ACC: Anterior Cingulate Cortex; CBF: Cerebral Blood Flow; Glx: Glutamate + Glutamine. * $\boldsymbol{P}<0.05$

## Table S6. Hierarchical regression of associations between baseline CBF and glutamate metabolites in the ACC and striatum prior to clozapine treatment and change in PANSS total scores (N=20).

|  | | | | | | | | | |
| --- | --- | --- | --- | --- | --- | --- | --- | --- | --- |
| **ACC** | **Model 1** | | | **Model 2** | | | **Model 3** | | |
|  |  | | |  | | |  | | |
|  |  | | |  | | |  | | |
| **Step 1: Covariates** | $\boldsymbol{B(\beta)}$ | $\boldsymbol{t}$ | $\boldsymbol{P}$ | $\boldsymbol{B(\beta)}$ | $\boldsymbol{t}$ | $\boldsymbol{P}$ | $\boldsymbol{B(\beta)}$ | $\boldsymbol{t}$ | $\boldsymbol{P}$ |
| Baseline global CBF | -0.93 (-0.43) | -2.03 | 0.06 | -2.11 (-0.98) | -0.96 | 0.35 | -1.78 (-0.83) | -0.77 | 0.45 |
| **Step 2: Predictors** |  |  |  |  |  |  |  |  |  |
| Baseline ACC CBF |  |  |  | 1.12 (0.55) | 0.54 | 0.60 | 3.60 (1.77) | 0.75 | 0.46 |
| Baseline ACC Glutamate |  |  |  | 3.93 (0.34) | 1.63 | 0.12 | 10.94 (0.95) | 0.88 | 0.39 |
| **Step 3: Interaction terms** |  |  |  |  |  |  |  |  |  |
| Baseline ACC CBF x Baseline ACC Glutamate |  |  |  |  |  |  | -0.19 (-1.54) | -0.58 | 0.57 |
| $\boldsymbol{R}$**^2^** | 0.19 | | | 0.33 | | | 0.34 | | |
| $\boldsymbol{\Delta R}$**^2^** |  | | | 0.14 | | | 0.01 | | |
| **F-statistic (df_1_, df_2_)** | 4.10 (1, 18) | | | 2.58 (3, 16) | | | 1.94 (4, 15) | | |
|  |  |  |  |  |  |  |  |  |  |
|  |  |  |  |  |  |  |  |  |  |
| **Step 1: Covariates** | $\boldsymbol{B(\beta)}$ | $\boldsymbol{t}$ | $\boldsymbol{P}$ | $\boldsymbol{B(\beta)}$ | $\boldsymbol{t}$ | $\boldsymbol{P}$ | $\boldsymbol{B(\beta)}$ | $\boldsymbol{t}$ | $\boldsymbol{P}$ |
| Baseline global CBF | -0.93 (-0.43) | -2.03 | 0.06 | -3.06 (-1.42) | -1.51 | 0.15 | -3.06 (-1.43) | -1.51 | 0.15 |
| **Step 2: Predictors** |  |  |  |  |  |  |  |  |  |
| Baseline ACC CBF |  |  |  | 1.97 (0.97) | 1.03 | 0.32 | 4.02 (1.98) | 1.39 | 0.19 |
| Baseline ACC Glx |  |  |  | 2.84 (0.45) | 2.31 | 0.03* | 6.10 (0.96) | 1.67 | 0.12 |
| **Step 3: Interaction terms** |  |  |  |  |  |  |  |  |  |
| Baseline ACC CBF x Baseline ACC Glx |  |  |  |  |  |  | -0.10 (-1.18) | -0.95 | 0.36 |
| $\boldsymbol{R}$**^2^** | 0.19 | | | 0.41 | | | 0.44 | | |
| $\boldsymbol{\Delta R}$**^2^** |  | | | 0.22 | | | 0.03 | | |
| **F-statistic (df_1_, df_2_)** | 4.10 (1, 18) | | | 3.72 (3, 16) * | | | 3.00 (4, 15) | | |
|  |  |  |  |  |  |  |  |  |  |
|  |  |  |  |  |  |  |  |  |  |
| **Striatum** | **Model 1** | | | **Model 2** | | | **Model 3** | | |
|  |  |  |  |  |  |  |  |  |  |
|  |  |  |  |  |  |  |  |  |  |
| **Step 1: Covariates** | $\boldsymbol{B(\beta)}$ | $\boldsymbol{t}$ | $\boldsymbol{P}$ | $\boldsymbol{B(\beta)}$ | $\boldsymbol{t}$ | $\boldsymbol{P}$ | $\boldsymbol{B(\beta)}$ | $\boldsymbol{t}$ | $\boldsymbol{P}$ |
| Baseline global CBF | -0.93 (-0.43) | -2.03 | 0.06 | -2.87 (-1.34) | -2.37 | 0.03* | -2.78 (-1.29) | -2.20 | 0.04* |
| **Step 2: Predictors** |  |  |  |  |  |  |  |  |  |
| Baseline Striatum CBF |  |  |  | 3.11 (1.01) | 1.74 | 0.10 | 5.03 (1.64) | 0.99 | 0.34 |
| Baseline Striatum Glutamate |  |  |  | -2.61 (-0.19) | -0.84 | 0.42 | 4.55 (0.33) | 0.26 | 0.80 |
| **Step 3: Interaction terms** |  |  |  |  |  |  |  |  |  |
| Baseline Striatum CBF x Baseline Striatum Glutamate |  |  |  |  |  |  | -0.18 (-0.95) | -0.41 | 0.69 |
| $\boldsymbol{R}$**^2^** | 0.19 | | | 0.32 | | | 0.33 | | |
| $\boldsymbol{\Delta R}$**^2^** |  | | | 0.13 | | | 0.01 | | |
| **F-statistic (df_1_, df_2_)** | 4.10 (1, 18) | | | 2.48 (3, 16) | | | 1.80 (4, 15) | | |
|  |  |  |  |  |  |  |  |  |  |
|  |  |  |  |  |  |  |  |  |  |
| **Step 1: Covariates** | $\boldsymbol{B(\beta)}$ | $\boldsymbol{t}$ | $\boldsymbol{P}$ | $\boldsymbol{B(\beta)}$ | $\boldsymbol{t}$ | $\boldsymbol{P}$ | $\boldsymbol{B(\beta)}$ | $\boldsymbol{t}$ | $\boldsymbol{P}$ |
| Baseline global CBF | -0.93 (-0.43) | -2.03 | 0.06 | -2.65 (-1.24) | -2.12 | 0.05 | -2.56 (-1.19) | -1.88 | 0.08 |
| **Step 2: Predictors** |  |  |  |  |  |  |  |  |  |
| Baseline Striatum CBF |  |  |  | 2.67(0.87) | 1.48 | 0.16 | 3.20 (1.04) | 0.98 | 0.34 |
| Baseline Striatum Glx |  |  |  | -0.25 (-0.04) | -0.17 | 0.87 | 1.55 (0.24) | 0.17 | 0.87 |
| **Step 3: Interaction terms** |  |  |  |  |  |  |  |  |  |
| Baseline Striatum CBF x Baseline Striatum Glx |  |  |  |  |  |  | -0.04 (-0.37) | -0.20 | 0.85 |
| $\boldsymbol{R}$**^2^** | 0.19 | | | 0.29 | | | 0.29 | | |
| $\boldsymbol{\Delta R}$**^2^** |  | | | 0.10 | | | 0.00 | | |
| **F-statistic (df_1_, df_2_)** | 4.10 (1, 18) | | | 2.16 (3, 16) | | | 1.53 (4, 15) | | |
|  |  |  |  |  |  |  |  |  |  |

Baseline global CBF was entered in step 1 (Model 1, control variable); Baseline glutamate metabolites and baseline CBF values as predictors in step 2, covarying for baseline global CBF (Model 2); The interaction term baseline glutamate metabolites x CBF values was entered in step 3 (Model 3).

$B$: Unstandardized coefficient; $\beta$: standardized beta coefficient; $R$^2^: proportion of variance in dependent variable explained by the model; $\Delta R$^2^: change in $R$^2^ from the previous step; df_1_: numerator degrees of freedom; df_2_: denominator degrees of freedom; F statistic: F-test of model. ACC: Anterior Cingulate Cortex; CBF: Cerebral Blood Flow; Glx: Glutamate + Glutamine. * $\boldsymbol{P}<0.05$

##

## Table S7. Hierarchical regression of associations between baseline CBF and glutamate metabolites in the ACC and striatum prior to clozapine treatment and change in PANSS total scores, including clozapine dose at week 12 (N=20).

|  | | | | | | | | | | | | |
| --- | --- | --- | --- | --- | --- | --- | --- | --- | --- | --- | --- | --- |
| **ACC** | **Model 1** | | | **Model 2** | | | **Model 3** | | | **Model 4** | | |
|  |  | | |  | | |  | | |  | | |
|  |  | | |  | | |  | | |  | | |
| **Step 1: Covariates** | $\boldsymbol{B(\beta)}$ | $\boldsymbol{t}$ | $\boldsymbol{P}$ | $\boldsymbol{B(\beta)}$ | $\boldsymbol{t}$ | $\boldsymbol{P}$ | $\boldsymbol{B(\beta)}$ | $\boldsymbol{t}$ | $\boldsymbol{P}$ | $\boldsymbol{B(\beta)}$ | $\boldsymbol{t}$ | $\boldsymbol{P}$ |
| Baseline global CBF | -0.80 (-0.38) | -1.68 | 0.11 | -2.60 (-1.23) | -1.15 | 0.27 | -2.21 (-1.05) | -0.96 | 0.35 | -1.15 (-0.54) | -0.52 | 0.61 |
| **Step 2: Predictors** |  |  |  |  |  |  |  |  |  |  |  |  |
| Baseline ACC CBF |  |  |  | 1.72 (0.84) | 0.79 | 0.44 | 6.41 (3.15) | 1.22 | 0.24 | 5.59 (2.75) | 1.14 | 0.27 |
| Baseline ACC Glutamate |  |  |  | 3.40 (0.31) | 1.36 | 0.19 | 15.75 (1.42) | 1.23 | 0.24 | 13.62 (1.23) | 1.14 | 0.27 |
| **Step 3: Interaction terms** |  |  |  |  |  |  |  |  |  |  |  |  |
| Baseline ACC CBF x Baseline ACC Glutamate |  |  |  |  |  |  | -0.35 (-2.85) | -0.98 | 0.34 | -0.35 (-2.85) | -1.06 | 0.31 |
| **Step 4: Clozapine dose** |  |  |  |  |  |  |  |  |  |  |  |  |
| Week 12 clozapine dose |  |  |  |  |  |  |  |  |  | 0.07 (0.44) | 1.83 | 0.09 |
| $\boldsymbol{R}$**^2^** | 0.14 | | | 0.29 | | | 0.34 | | | 0.47 | | |
| $\boldsymbol{\Delta R}$**^2^** |  | | | 0.15 | | | 0.05 | | | 0.13 | | |
| **F-statistic (df_1_, df_2_)** | 2.83 (1, 17) | | | 2.04 (3, 15) | | | 1.77 (4, 14) | | | 2.32 (5, 13) | | |
|  |  |  |  |  |  |  |  |  |  |  |  |  |
|  |  |  |  |  |  |  |  |  |  |  |  |  |
| **Step 1: Covariates** | $\boldsymbol{B(\beta)}$ | $\boldsymbol{t}$ | $\boldsymbol{P}$ | $\boldsymbol{B(\beta)}$ | $\boldsymbol{t}$ | $\boldsymbol{P}$ | $\boldsymbol{B(\beta)}$ | $\boldsymbol{t}$ | $\boldsymbol{P}$ | $\boldsymbol{B(\beta)}$ | $\boldsymbol{t}$ | $\boldsymbol{P}$ |
| Baseline global CBF | -0.80 (-0.38) | -1.68 | 0.11 | -3.35 (-1.58) | -1.61 | 0.13 | -3.72 (-1.76) | -1.87 | 0.08 | -2.65 (-1.26) | -1.22 | 0.24 |
| **Step 2: Predictors** |  |  |  |  |  |  |  |  |  |  |  |  |
| Baseline ACC CBF |  |  |  | 2.36 (1.16) | 1.18 | 0.26 | 6.80 (3.34) | 2.05 | 0.06 | 5.32 (2.62) | 1.52 | 0.15 |
| Baseline ACC Glx |  |  |  | 2.58 (0.42) | 1.99 | 0.06 | 8.56 (1.38) | 2.22 | 0.04* | 6.71 (1.08) | 1.63 | 0.13 |
| **Step 3: Interaction terms** |  |  |  |  |  |  |  |  |  |  |  |  |
| Baseline ACC CBF x Baseline ACC Glx |  |  |  |  |  |  | -0.19 (-2.40) | -1.64 | 0.12 | -0.16 (-2.04) | -1.38 | 0.19 |
| **Step 4: Clozapine terms** |  |  |  |  |  |  |  |  |  |  |  |  |
| Week 12 clozapine dose |  |  |  |  |  |  |  |  |  | 0.05 (0.29) | 1.16 | 0.27 |
| $\boldsymbol{R}$**^2^** | 0.14 | | | 0.37 | | | 0.47 | | | 0.52 | | |
| $\boldsymbol{\Delta R}$**^2^** |  | | | 0.23 | | | 0.10 | | | 0.05 | | |
| **F-statistic (df_1_, df_2_)** | 2.83 (1, 17) | | | 2.93 (3, 15) | | | 3.11 (4, 14) | | | 2.82 (5, 13) | | |
|  |  |  |  |  |  |  |  |  |  |  |  |  |
|  |  |  |  |  |  |  |  |  |  |  |  |  |
| **Striatum** | **Model 1** | | | **Model 2** | | | **Model 3** | | | **Model 4** | | |
|  |  |  |  |  |  |  |  |  |  |  |  |  |
|  |  |  |  |  |  |  |  |  |  |  |  |  |
| **Step 1: Covariates** | $\boldsymbol{B(\beta)}$ | $\boldsymbol{t}$ | $\boldsymbol{P}$ | $\boldsymbol{B(\beta)}$ | $\boldsymbol{t}$ | $\boldsymbol{P}$ | $\boldsymbol{B(\beta)}$ | $\boldsymbol{t}$ | $\boldsymbol{P}$ | $\boldsymbol{B(\beta)}$ | $\boldsymbol{t}$ | $\boldsymbol{P}$ |
| Baseline global CBF | -0.80 (-0.38) | -1.68 | 0.11 | -2.97 (-1.41) | -2.54 | 0.02* | -2.82 (-1.33) | -2.34 | 0.03* | -1.58 (-0.75) | -1.39 | 0.19 |
| **Step 2: Predictors** |  |  |  |  |  |  |  |  |  |  |  |  |
| Baseline Striatum CBF |  |  |  | 3.59 (1.18) | 2.05 | 0.06 | 7.23 (2.38) | 1.44 | 0.17 | 6.15 (2.02) | 1.44 | 0.17 |
| Baseline Striatum Glutamate |  |  |  | -3.71 (-0.28) | -1.20 | 0.25 | 9.46 (0.71) | 0.55 | 0.59 | 9.54 (0.72) | 0.65 | 0.53 |
| **Step 3: Interaction terms** |  |  |  |  |  |  |  |  |  |  |  |  |
| Baseline Striatum CBF x Baseline Striatum Glutamate |  |  |  |  |  |  | -0.34 (-1.86) | -0.78 | 0.45 | -0.37 (-2.05) | -1.01 | 0.33 |
| **Step 4: Clozapine terms** |  |  |  |  |  |  |  |  |  |  |  |  |
| Week 12 clozapine dose |  |  |  |  |  |  |  |  |  | 0.09 (0.53) | 2.53 | 0.03* |
| $\boldsymbol{R}$**^2^** | 0.14 | | | 0.34 | | | 0.36 | | | 0.57 | | |
| $\boldsymbol{\Delta R}$**^2^** |  | | | 0.20 | | | 0.02 | | | 0.21 | | |
| **F-statistic (df_1_, df_2_)** | 2.83 (1, 17) | | | 2.54 (3, 15) | | | 2.01 (4, 14) | | | 3.50 (5, 13) * | | |
|  |  |  |  |  |  |  |  |  |  |  |  |  |
|  |  |  |  |  |  |  |  |  |  |  |  |  |
| **Step 1: Covariates** | $\boldsymbol{B(\beta)}$ | $\boldsymbol{t}$ | $\boldsymbol{P}$ | $\boldsymbol{B(\beta)}$ | $\boldsymbol{t}$ | $\boldsymbol{P}$ | $\boldsymbol{B(\beta)}$ | $\boldsymbol{t}$ | $\boldsymbol{P}$ | $\boldsymbol{B(\beta)}$ | $\boldsymbol{t}$ | $\boldsymbol{P}$ |
| Baseline global CBF | -0.80 (-0.38) | -1.68 | 0.11 | -2.81 (-1.33) | -2.29 | 0.04* | -2.58 (-1.22) | -1.94 | 0.07 | -1.52 (-0.72) | -1.15 | 0.27 |
| **Step 2: Predictors** |  |  |  |  |  |  |  |  |  |  |  |  |
| Baseline Striatum CBF |  |  |  | 3.19 (1.05) | 1.76 | 0.10 | 4.77 (1.57) | 1.42 | 0.18 | 3.00 (0.99) | 0.94 | 0.37 |
| Baseline Striatum Glx |  |  |  | -0.90 (-0.14) | -0.60 | 0.56 | 4.16 (0.65) | 0.46 | 0.66 | 2.76 (0.43) | 0.33 | 0.75 |
| **Step 3: Interaction terms** |  |  |  |  |  |  |  |  |  |  |  |  |
| Baseline Striatum CBF x Baseline Striatum Glx |  |  |  |  |  |  | -0.12 (-1.10) | -0.56 | 0.58 | -0.09 (-0.85) | -0.47 | 0.64 |
| **Step 4: Clozapine terms** |  |  |  |  |  |  |  |  |  |  |  |  |
| Week 12 clozapine dose |  |  |  |  |  |  |  |  |  | 0.07 (0.46) | 1.96 | 0.07 |
| $\boldsymbol{R}$**^2^** | 0.14 | | | 0.29 | | | 0.31 | | | 0.47 | | |
| $\boldsymbol{\Delta R}$**^2^** |  | | | 0.15 | | | 0.02 | | | 0.16 | | |
| **F statistic (df_1_, df_2_)** | 2.83 (1, 17) | | | 2.05 (3, 15) | | | 1.54 (4, 14) | | | 2.26 (5, 13) | | |
|  |  |  |  |  |  |  |  |  |  |  |  |  |

Baseline global CBF was entered in step 1 (Model 1, control variable); Baseline glutamate metabolites and baseline CBF values as predictors in step 2, covarying for baseline global CBF (Model 2); The interaction term baseline glutamate metabolites x CBF values was entered in step 3 (Model 3); Week 12 clozapine dose was entered as covariates (Model 4).

$B$: Unstandardized coefficient; $\beta$: standardized beta coefficient; $R$^2^: proportion of variance in dependent variable explained by the model; $\Delta R$^2^: change in $R$^2^ from the previous step; df_1_: numerator degrees of freedom; df_2_: denominator degrees of freedom; F statistic: F-test of model. ACC: Anterior Cingulate Cortex; CBF: Cerebral Blood Flow; Glx: Glutamate + Glutamine. * $\boldsymbol{P}<0.05$

# Supplementary Figures

## Figure S1. Representative images of ^1^H-MRS voxel placement the anterior cingulate cortex (A) and striatum (B).


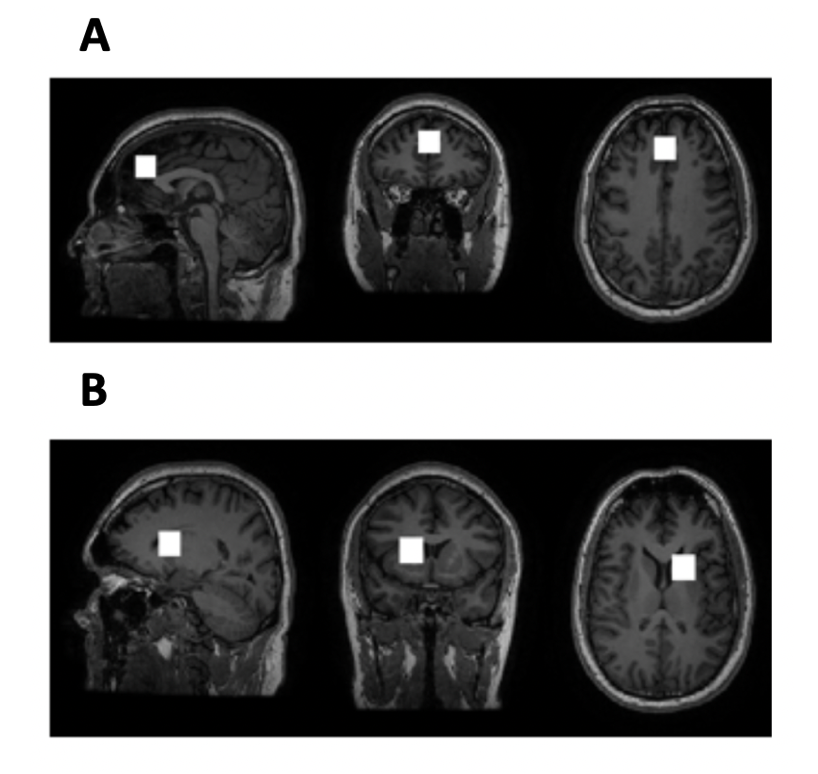


## Figure S2. Striatal glutamate before and after 12 weeks of clozapine treatment (N=20).


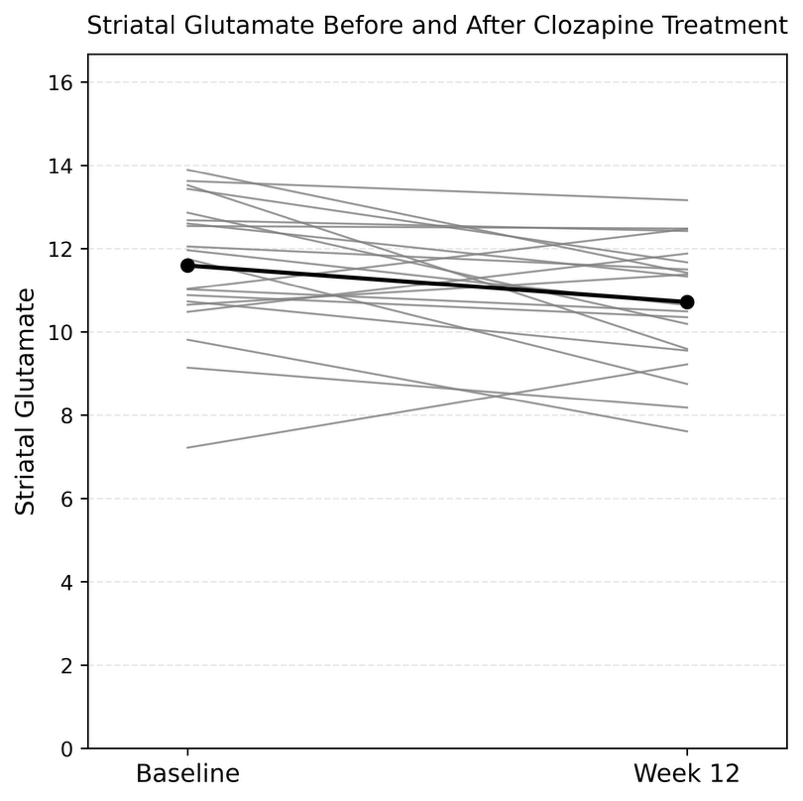


Individual trajectories are represented by grey lines. Mean values (black circles) are displayed, with the black line depicting a significant decrease in striatal glutamate from baseline to week 12 ($p$ = 0.02).
